# Editorial: Bioengineering and Biotechnology Approaches in Cardiovascular Sciences

**DOI:** 10.3389/fbioe.2021.746435

**Published:** 2021-08-20

**Authors:** Jianyi Zhang, Martin L. Tomov, James J. H. Chong, Philippe Menasché, Vahid Serpooshan

**Affiliations:** ^1^ Department of Biomedical Engineering, School of Medicine and School of Engineering, University of Alabama at Birmingham, Birmingham, AL, United States; ^2^ Division of Cardiovascular Diseases, Department of Medicine, School of Medicine, University of Alabama at Birmingham, Birmingham, AL, United States; ^3^ Department of Biomedical Engineering, Emory University School of Medicine and Georgia Institute of Technology, Atlanta, GA, United States; ^4^ Sydney Medical School, The University of Sydney, Sydney, NSW, Australia; ^5^ Centre for Heart Research, Westmead Institute for Medical Research, The University of Sydney, Sydney, NSW, Australia; ^6^ Department of Cardiology, Westmead Hospital, Sydney, NSW, Australia; ^7^ Department of Cardiovascular Surgery, Hôpital Européen Georges Pompidou, Université de Paris, PARCC, INSERM, Paris, France; ^8^ Department of Pediatrics, Emory University School of Medicine, Atlanta, GA, United States; ^9^ Children’s Healthcare of Atlanta, Atlanta, GA, United States

**Keywords:** bioengineering, biotechnologhy, cardiovascular medicine, tissue engieering, stem cell therapies

Medical science has often looked to the fields of bioengineering and biotechnology as tools that can facilitate the clinical translation of new therapeutic strategies. This Research Topic, *Bioengineering and Biotechnology Approaches in Cardiovascular Sciences*, focuses on cardiovascular bioengineering principles that seek to develop cells and tissues that fully recapitulate the functional properties of their native analogs. Early studies were frequently limited by a lack of cells, especially cardiomyocytes (CMs), but progress has accelerated since the development of techniques for reprogramming somatic cells into induced pluripotent stem cells (iPSCs) and then differentiating them into nearly any desired lineage. Here, we present a collection of 13 original research and review articles that provide the reader with a broad overview of recent discoveries and innovations that may expedite the use of bioengineered cells and tissues for therapeutic applications, disease modeling, and drug testing.

## Cardiac Therapy

With pluripotent cells and their differentiated progeny beginning to be investigated in patients, Menasché has reviewed their use from a clinical perspective. The number of patients enrolled in these early-phase trials has been too low to conclusively evaluate the efficacy of treatment, but concerns regarding the potential for treatment-related oncogenesis are largely resolved, and no other safety issues have been observed. The author also notes that most of the improvements associated with cell transplantation can be replicated *via* administration of the products that the cells produce and secrete in culture, and that the practical benefits of secretome administration could be advantageous for clinical applications. Furthermore, some evidence suggests that much of the benefit of myocardial cell therapy may be attributable to an acute immune response; Elde et al. briefly discuss how this new insight intersects with recent innovations in molecular and cell biology, biomaterials, biomechanics, and tissue engineering.

Much of the work in regenerative myocardial therapy has focused on CMs—the fundamental contractile units of the heart—and on the vascular cells that support them. However, Chen et al. discuss how cardiac fibroblasts (CFs) may contribute to myocardial regeneration by inducing CM proliferation and modulating the stiffness of the extracellular matrix. Notably, iPSCs retain many of the epigenetic characteristics present in the cells from which they were reprogrammed. Thus, iPSCs generated from CFs may be more suitable for cardiac therapy than those produced from other cell lineages. Conventional methods for iPSC-CM differentiation have been conducted in culture plates with two-dimensional (2D) cell sheets, but Kahn-Krell et al. introduce a three-dimensional (3D) differentiation protocol that is more suitable for commercial production. Their approach yields up to 1.5 million cells/mL, with an efficiency of greater than 98% and lower between-batch variability than traditional 2D protocols.

CFs have also been reprogrammed directly into CMs without passing through an intermediate iPSC stage, which suggests that the endogenous pool of CFs could serve as a source of new CMs for remuscularizing damaged myocardium. Riching and Song provide an in-depth review of direct CF-to-CM reprogramming, including the endogenous signaling pathways and epigenetic markers that influence reprogramming efficiency, as well as less extensive discussions of therapeutic revascularization and the transient induction of CM proliferation. Both direct and indirect reprogramming are discussed by Chingale et al., who also review the use of viral and non-viral vectors, methods for targeting reprogramming vectors and therapeutics to the heart, and how cell-derived proteins, exosomes, and microRNAs have been used to overcome limitations in cell production. They also highlight recent advancements in the manufacturing of cardiac patches *via* 3D bioprinting.

Cardiac function can also be compromised by disruptions in heart rhythm, and although electronic pacemakers can be life-saving, they cannot adapt to somatic growth or respond to physiological changes in the autonomic nervous system. Thus, this collection of articles also includes a report by Naumova and Lop, who review strategies for applying the principles of bioengineering and biotechnology to the development of biological pacemakers.

## Tissue Engineering

Many engineered tissues are produced by suspending cells within a biomaterial scaffold, but ensuring that the cells are homogenously distributed throughout the 3D scaffold can be challenging for constructs of clinically relevant sizes. Cunnane et al. have developed a semi-automated seeding device that can homogenously distribute up to 200 million cells along both the length and circumference of 10-cm long tissue-engineered vascular grafts (TEVGs). When transplanted into the carotid arteries of sheep, mechanical and histological assessments conducted 10 weeks later confirmed graft patency and indicated that the TEVG was being gradually remodeled with the native tissue.

Engineered tissue transplants are frequently secured in place with sutures or bioglues, which may fail to provide adequate adhesion strength or induce secondary damage, cytotoxicity, and adverse immune reactions. Chen et al. explore the mechanisms that influence transplant adhesion and then highlight the development of scaffolds that are intrinsically adhesive [i.e., adhesive tissue engineered scaffolds (ATESs)]. They also discuss some of the key challenges associated with the use of ATES in specific tissue engineering applications and strategies for both qualitatively and quantitatively monitoring adhesion. Furthermore, the integrity and function of transplanted tissues are crucially dependent on adequate vascularization. Although skeletal muscle myoblasts (SMMs) have been extensively studied for treatment of cardiac injury with little evidence of myocardial regeneration, they appear to improve recovery and limit remodeling by secreting cytokines that promote angiogenesis. Skeletal muscle fibroblasts (SMFs) also secrete pro-angiogenic cytokines. Thummarati and Kino-Oka demonstrate that the formation of endothelial networks in multilayered sheets of human SMMs and SMFs was greatest when 30–40% of the cells were SMFs. Thus, combinations of human SMFs and SMMs may improve the vascularity and transplantation efficiency of engineered tissues.

## Preclinical and *In Vitro* Modeling

The clinical translation of new pharmaceutical and cell-based treatments is crucially dependent on the development of appropriate animal models. Shin et al., review the challenges associated with modeling the complexity of human ischemic cardiovascular disease in large animals, which include not only the anatomical and physiological differences between animals and humans, but also species-specific surgical modifications, limitations in sample size, and a lack of sufficient transparency in published reports. Large animal studies are also typically conducted in young, healthy animals and, consequently, fail to replicate the variations in age, comorbidity, and overall health observed in patients.

Because the clinical implications of observations in animals are limited by fundamental differences between humans and the model species, the results from animal studies are best interpreted in concert with those from experiments performed with engineered human tissue and organoid chips. These technologies are more physiologically relevant than conventional cell-culture models, because they are designed to replicate key properties of the native environment, including interactions among multiple cell types, mechanical stress, and other dynamic factors. Furthermore, since iPSCs can be differentiated from the somatic cells of each individual patient, chips constructed with iPSC-derived cells could be powerful tools for personalizing patient diagnosis and treatment. Chan and Huang review the design and construction of tissue chips and their application as models for studying the pathogenesis of cardiovascular disease and drug testing, while Zhao et al. discuss organoids and organoid chips, which mimic the even more complex structural and functional properties of entire organs. Organoid chips have already been used to model the lung, kidney, and blood-brain barrier, but the functional and structural integrity of organoid models may be compromised by deficiencies in vascularity; thus, the authors include a detailed discussion of techniques such as 3D and 4D (i.e., 3D plus time) bioprinting that can be used to generate more extensive and sophisticated vascular networks.

## Summary

The articles presented in this Research Topic provide a synopsis of many of the most recent advancements in cardiovascular cell and tissue bioengineering. The Editors are pleased to highlight this work and hope that it may help provide a foundation for future studies that could lead to the development of transformative new investigational tools and therapeutic approaches in the field of cardiovascular medicine.

